# Difference in oral microbial composition between chronic periodontitis patients with and without diabetic nephropathy

**DOI:** 10.1186/s12903-021-01985-3

**Published:** 2022-01-16

**Authors:** Dongxue Zhang, Wenyan Liu, Li Peng, Haiyan Wang, Mei Lin, Yufeng Li, Zuomin Wang

**Affiliations:** 1 Department of Stomatology, Beijing Chao-Yang Hospital, Capital Medical University, No. 8 South Gongti Road, Chao Yang District, Beijing, 100020 China; 2 Department of Stomatology, Beijing Luhe Hospital, Capital Medical University, Beijing, 101149 China; 3 Department of Stomatology, The Third People’s Hospital of Datong City, Datong, 037008 China; 4 Department of Endocrinology, Beijing Friendship Hospital Pinggu Campus, Capital Medical University, No.59 Xinping North Road, Pinggu District, Beijing, 101200 China

**Keywords:** Diabetic nephropathy, Diabetes mellitus, Periodontitis, Saliva, Oral microbiome

## Abstract

**Background:**

To investigate the difference in the structural composition of salivary flora between chronic periodontitis patients with and without diabetic nephropathy (DN).

**Methods:**

Thirty salivary samples of 15 chronic periodontitis patients with DN (DN group) and 15 chronic periodontitis patients with diabetes but without DN (DM group) were subjected to pyrosequencing of polymerase chain reaction-amplified 16 s ribosomal RNA genes. After diversity testing, the differential flora were analyzed. The sequencing results were compared with GenBank database to determine the type of differential flora using species composition analysis, hierarchical cluster analysis, principal co-ordinate analysis, and species difference analysis.

**Results:**

There were significant between-group differences with respect to *Gemella*, *Selenomonas spp*, *Lactobacillales_unclassified, Bacteria-unclassified* and *Abiotrophia* (*p* < 0.05). Compared with DM group, the relative abundance of *Selenomonas spp.* in DN group was significantly higher; the area under the receiver operating characteristic curve of *Selenomonas spp.* was 0.713 (*P* < 0.05). Multi-level biological identification and feature maps indicated that *Selenomonas spp.* might be used as a potential biomarker for DN patients. On binary logistic regression analysis, increase of *Selenomonas spp.* was related with DN.

**Conclusions:**

We found significant between-group differences in the structural composition of oral flora. The increase in the relative abundance of *Selenomonas spp.* may be associated with DN in patients with chronic periodontitis.

**Supplementary Information:**

The online version contains supplementary material available at 10.1186/s12903-021-01985-3.

## Background

Chronic periodontitis (CP) is a chronic infectious disease caused by plaque microorganisms; the severity of CP is associated with systemic inflammation[1]. Previous studies have demonstrated the bidirectional relationship between periodontitis and diabetes mellitus [2]. Patients with type 2 diabetes mellitus (T2DM) have a greater risk of periodontitis onset and progression than healthy patients [3], while periodontitis adversely affects glycemic control in diabetic patients and contributes to the development of diabetic complications [4]. These findings indicate that periodontitis and diabetes mellitus can affect each other.

Several studies have demonstrated the relationship between periodontitis and the occurrence of several systemic diseases; this relationship may be mediated via inflammatory pathways and changes in oral flora. The oral microbiome not only contributes to the human oral diseases but has also been considered as a significant risk factor for various diseases, such as diabetes mellitus [5]. The α- and β-diversity of the salivary microbiome were significantly different between the nondiabetic periodontitis patients and patients with a history of diabetes [6], while the β-diversity difference of the salivary microbiome was not related to the severity of periodontitis. Moreover, diabetes mellitus was found to significantly alter the salivary microbiota of periodontitis patients, while treatment did not lead to flora recovery [6]. However, although another study also found a greater diversity of saliva microbiota in diabetic periodontitis patients than in nondiabetic periodontitis patients, hypoglycemic therapy could reconstruct the saliva microbiota and hence improve the localized conditions of diabetes patients with periodontitis [7]. In a study, diabetes and pre-diabetes patients showed reduced biological and phylogenetic diversity of the oral microbiota compared with healthy people [8]. In another study, oral microbial diversity was shown to decrease in diabetic periodontitis patients, and increase with the progression of periodontal disease [9]. These studies suggest that oral microbiota are related with both periodontitis and diabetes.

Previous studies have also reported the association between periodontitis and chronic kidney disease (CKD) [10]. Patients with CKD have an increased incidence of periodontitis. Hypertension and the duration of diabetes are important factors that may affect the two-way relationship between chronic periodontitis and CKD [11]. Diabetic nephropathy (DN) is one of the main microvascular complications of diabetes mellitus; it is also one of the main causes of end-stage renal disease (ESRD) [12]. In a recent study, chronic periodontitis was found to be a risk factor for renal dysfunction in DM patients [13]. Zhang et al. compared the microbial flora of periodontitis patients with or without peritoneal dialysis; they found that *Prevotellaceae, Selenomonas, Aggregatibacter, Anaeroglobus, TM7_[G-5]*, and *Centipeda* were significantly enriched in the subgingival flora of peritoneal dialysis patients with periodontitis [14]. However, whether the oral microbiome changes during the progression of periodontitis patients with T2DM to DN remains unclear.

The aim of this pilot study was to examine the differences of salivary microbiome between patients with periodontitis and T2DM with or without DN. Our findings may help identify whether changes in some specific bacteria or salivary microbiome are associated with the development of DN and periodontitis.

## Methods

### Subject selection and grouping

This was an observational study conducted from July to August 2017. A total of 30 T2DM patients (with or without DN) who had CP were recruited from the outpatient section of the Department of Medicine, Pinggu Hospital, Beijing, China. The inclusion criteria were: (1) type 2 diabetes mellitus with or without renal complications; (2) age range, 30–75 years; (3) presence of at least 5 existing natural teeth; (4) provision of written informed consent for participation.

The exclusion criteria were: (1) pregnant or lactating women; (2) patients with type 1 diabetes; (3) history of periodontal treatment within the preceding 6 months; (4) intake of anti-inflammatory drugs and/or antibiotics within the preceding 3 months.

The diagnosis of T2DM was established according to the 2011 guidelines of the American Diabetic Association (ADA) [15]. The diagnosis of DN was established according to the American Diabetic Association (ADA, 2014) and National Kidney Foundation [16] guidelines. The diagnosis of CP was based on the standards of the 1999 International Workshop for Classification of Periodontal Disease and Conditions [17].

Patients were categorized into two groups: 15 patients with CP and T2DM (DM group); 15 patients with CP and DN (DN group). Each group consisted of 5 patients with mild periodontitis, 5 patients with moderate periodontitis, and 5 patients with severe periodontitis. Patients in the two groups were matched with respect to age, sex, body mass index (BMI), fasting blood glucose (FBG), glycated hemoglobin (HbA1c), and status of periodontitis. This study was approved by the Ethics Committee of the Pinggu Hospital (No.: 2020–008-01).

### Periodontal examination

A specialized dentist carried out the clinical oral examinations. The number of teeth was recorded. Clinical examination consisted of bleeding index (BI), pocket probing depth (PD), clinical attachment loss (CAL), and remaining teeth number. A periodontal probe (Hu-Firedy, USA) was used for probe examination of each tooth (at six sites per tooth); the unit of measurement was millimeters. The periodontal probe was used to examine the cemento-enamel junction (CEJ) in all teeth, including the third molar. Recession was recorded as the distance from the free gingival margin to the CEJ. PD was the distance from the gingival margin to the bottom of the pocket. CAL was calculated as the sum of PD and distance from the gingival margin to the CEJ. The BI was scored on a scale of 0–5 [18]. According to the World Health Organization (WHO) survey methods for basic oral health, in order to determine the periodontitis classification [17], each site was air-dried before periodontal examination; the examiner probed the periodontal pocket with an intensity of ≤ 25 g. The classification [19] and diagnostic criteria for periodontitis were: mild periodontitis, at least one tooth with a PD ≥ 3 mm and CAL ≥ 3 mm, while PD ≥ 4 mm and CAL ≥ 3 mm ≤ 30%; moderate periodontitis, PD ≥ 5 mm and CAL ≥ 4 mm teeth < 30%, or teeth with PD ≥ 4 mm and CAL ≥ 3 mm between 30 to 60%; and severe periodontitis, PD ≥ 5 mm and CAL ≥ 4 mm teeth ≥ 30%; or PD ≥ 4 mm and CAL ≥ 3 mm teeth ≥ 60%. Original data of periodontal examination are shown in Additional file 1: Table S1.

**Table 1:** Clinical characteristics of the study population

	DM group	DN group	*P* value
Age, years	53.80 ± 12.08	58.00 ± 10.62	0.321
Male, n (%)	11 (73.33%)	7 (46.67%)	0.136
BMI (kg/m^2^)	26.25 ± 3.07	27.65 ± 4.88	0.356
Diabetes course (years)	7.20 ± 5.81	13.98 ± 6.98	0.007**
Smoking, n (%)	8 (52%)	6 (40%)	0.464
Biochemical parameters			
Urea nitrogen (mmol/L)	5.76 (4.75, 6.77)	7.27 (4.86, 9.14)	0.181
Uric acid (μmol/L)	285.01 ± 101.90	390.53 ± 61.10	0.002**
FBG (mmol/L)	9.46 ± 2.58	9.87 ± 3.49	0.712
HbA1c (%)	8.31 ± 2.27	7.99 ± 1.29	0.639
Urinary albumin (mg/L)	6.30 (5.00, 12.00)	48.60 (28.40, 293.40)	< 0.001**
Urinary creatinine (μmol/L)	53.10 (37.20, 90.50)	63.90 (32.20, 84.60	0.925
ACR (mg/g)	13.12 (7.79, 25.64)	78.98 (38.55, 354.61)	0.001**
eGFR (ml/L)	113.21 ± 24.25	85.51 ± 43.13	0.042*
Parameters of periodontitis			
PD (mm)	3.38 ± 0.95	3.37 ± 0.93	0.995
BI	3.31 ± 0.68	2.99 ± 0.70	0.377
CAL (mm)	4.64 ± 1.60	5.09 ± 2.00	0.216
Remaining teeth numbers	24.67 ± 4.98	22.87 ± 5.96	0.377

*P*-values calculated using two independent samples *t* test, Mann–Whitney U test, or Chi-squared test. P < 0.05 was statistically significant. *P < 0.05, **P < 0.01

BMI, body mass index; LDL, low density lipoprotein; HDL, high density lipoprotein; FBG, fasting blood glucose; HbA1c, glycated hemoglobin; ACR, albumin creatinine ratio; eGFR, estimated glomerular filtration rate; PD, probing depth; BI, blooding index; CAL, clinical attachment loss

### Physical and laboratory examination

Body mass index was calculated using the formula: weight (kg)/height (m)^2^. For blood investigations, venous blood samples were drawn in the morning after 12-h fasting and sent to the Clinical Analysis Laboratory of the Pinggu Hospital for assay. Biochemical tests [urea nitrogen, uric acid, and Fasting blood glucose (FBG)] were assayed by colorimetry (Seimens Advia 2400, Germany). Glycated hemoglobin (HbA1c, %) levels were determined using high-performance liquid chromatography (Tosoh G8, Tokyo, Japan). Fasting first-void morning spot urine samples were collected for testing of creatinine and urine albumin (Siemens-Bayer). Urinary albumin/creatinine ratio (ACR) was also calculated. The formula for calculation of estimated glomerular filtration rate (eGFR) was [140-age(years)] × weight (kg)/ [72 × serum creatinine (mg/dL)] (for female patients, the result was multiplied by 0.85).

### Sample collection

At the beginning of the oral examination, non-stimulated whole saliva sample (at least 5 mL) was collected; for blood investigations, whole blood samples (at least 2 mL) were collected and centrifuged at 3000 rpm for 10 min.

### DNA was extracted from saliva samples and amplified by PCR

The E.Z.N.A.® soil DNA Kit (Omega Bio-tek, U.S.) was used to extract microbial DNA from human saliva samples, according to the manufacturer’s protocol. The final DNA concentration and purification was determined using NanoDrop 2000 UV–vis spectrophotometer (Thermo Scientific, Wilmington, USA). Finally, DNA quality was checked using 1% agarose gel electrophoresis. The V3-V4 hypervariable regions of the bacteria 16S rRNA gene were amplified with primers 338F (5’-ACTCCTACGGGAGGCAGCAG-3’) and 806R (5’-GGACTACHVGGGTWTCTAAT-3’) using thermocycler PCR system (GeneAmp 9700, USA). We designed the PCR reaction process according to the primer study[20]. PCR reactions were performed in triplicate: 20 μL mixture, which contained 4 μL of 5 × FastPfu buffer, 2 μL of 2.5 mM dNTPs, 0.8 μL of each primer (5 μM), 0.4 μL of FastPfu polymerase, and 10 ng of template DNA. Based on the protocol of manufacturer, 2% agarose gel was used to extract the resulting PCR products using the AxyPrep DNA Gel Extraction Kit (Axygen Biosciences, USA) and quantified using QuantiFluor™-ST (Promega, USA) for further purification.

### Illumina MiSeq sequencing

We referred to the standard procedures of Majorbio Bio-Pharm Technology Co. Ltd. (Shanghai, China); the purified amplicons were equimolar combined and paired-end sequenced (2 × 300) on an Illumina MiSeq platform (Illumina, USA). The raw reads were deposited in the NCBI Sequence Read Archive (SRA) database (SUB7267456, PRJNA625173).

### Processing of sequencing data

The Raw fastq files were split into multiple files, quality-filtered with Trimmomatic, and merged with FLASH. The criteria were as follows: (i) Truncate reads would be cut in case of any site with an average quality score < 20 over a 50 bp sliding window. (ii) Primers were exactly matched; reads of ambiguous bases were deleted allowing for 2 nucleotide mismatches. (iii) We merged overlap sequences that were longer than 10 bp. Silva (Release128 http://www.arb-silva.de) was used to cluster operational taxonomic units (OTUs) with 97% similarity cutoff; UCHIME was used to identify and remove chimeric sequences. The RDP Classifier algorithm (http://rdp.cme.msu.edu/) against the Silva (SSU123) 16S rRNA database was used to analyze taxonomy of each 16S rRNA, with a confidence threshold of 70%.

### Statistical analysis

Data pertaining to clinical parameters were analyzed using statistical software (SPSS v 19.0; SPSS IBM Inc., USA). Normally distributed continuous variables are presented as mean ± standard deviation (SD), while non-normally distributed continuous variables are presented as median and interquartile range. Between-group differences were assessed using the independent *t*-test or Mann–Whitney U test. Categorical variables are presented as frequency (%) and between-group differences assessed using the Chi-squared test. Association between bacteria taxa and diabetic nephropathy was identified through ordinal regression analyses. We used QIIME1 (v1.8.0) software for bioinformatics analysis. Alpha-diversity estimates were computed; Ace, Chao, Shannon and Simpson were calculated based on the gene profile for each sample [21]. Based on Bray–Curtis distances at the OTU level, Principle Coordinate Analysis (PCoA) were used to perform beta (between sample) diversity analysis [22]. R language tool statistics and drawing were used to make Venn diagram in order to define the core microbiome at the species level [23]. Linear discriminant analysis Effect Size (LEfSe) was used to identify the species that most likely explained the significant differences between the two groups [24]. Since the present study focused on the relationship between the oral flora and DN in patients with chronic periodontitis, *Gemella* and *Selenomonas spp* were included in the logistic regression analysis. Multivariate logistic analysis adjusting the effect of age, gender, and BMI was also performed. The effect of differential flora on the development of DN was assessed using receiver operating characteristic (ROC) curve analysis. The value associated with the largest Youden’s index was set as the cutoff level. The Two-sample Test method of PICRUSt + STAMP combined with Kyoto Encyclopedia of Genes and Genomes (KEGG) were used to speculate the composition of functional genes in the sample. Two-sided *P* values < 0.05 were considered indicative of statistical significance.

## Results

### Characteristics of the study population

A total of 169 DM patients including 132 DN patients were enrolled. After matching for age, sex, BMI, FBG, HbA1c, and status of periodontitis, only 30 patients were included in the final analysis (Additional file 1: Fig. S1). The 30 patients (18 males and 12 females; mean age: 53.8 years) were categorized into DM group (n = 15) or DN group (n = 15). The mean disease course in the DN group was significantly longer than that in the DM group (13.98 ± 6.98 *vs.* 7.20 ± 5.81 years, *P* = 0.007). Compared with the DM group, the DN group had significantly lower levels of uric acid, urinary albumin, and eGFR, and higher levels of ACR (*P* < 0.05). Periodontal status was similar in the two groups; there were no significant between-group differences with respect to PD, BI, or the number of remaining teeth (Table 1).

**Fig. 1:**
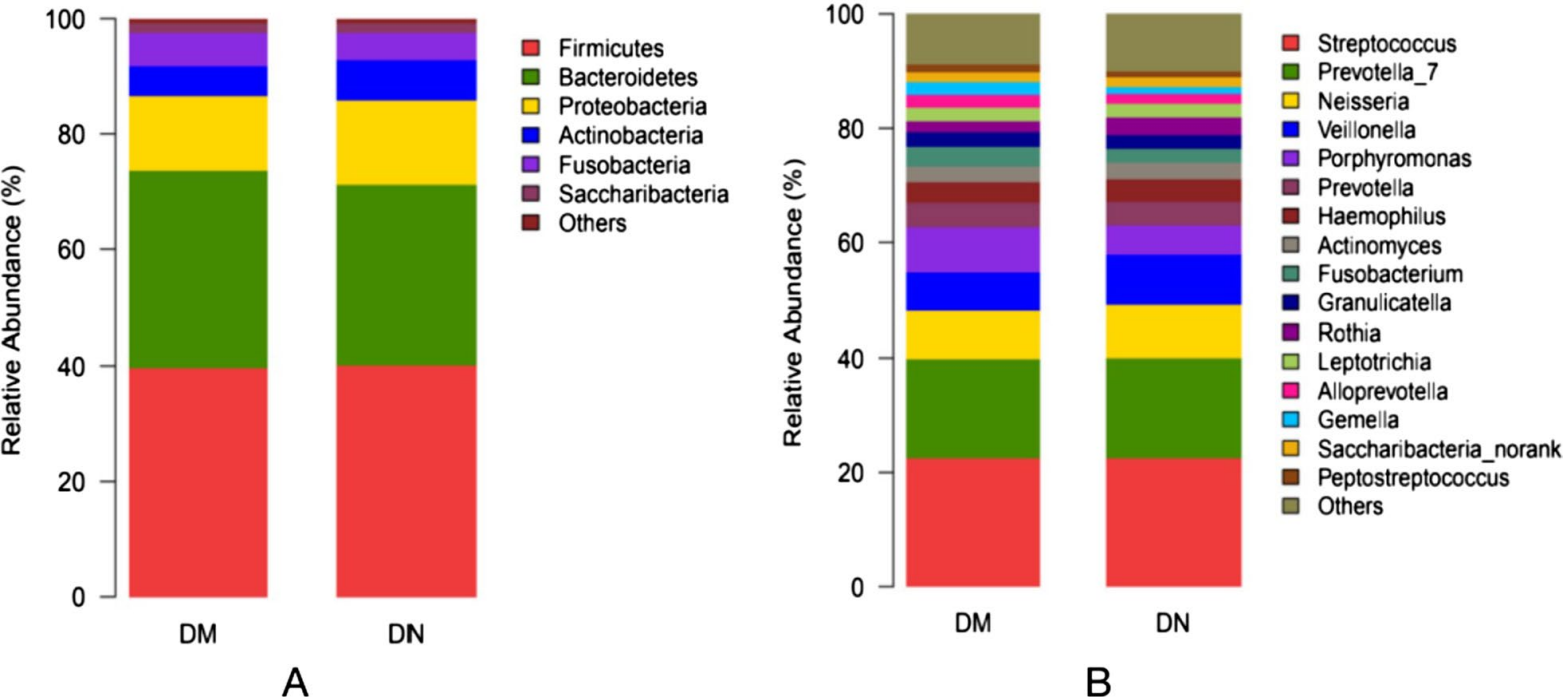
Analysis of oral microbiota composition in the study population (from phylum [**a**] to genus [**b**] level). For better visualization of the effect, parts with abundance < 1% have been merged and displayed in the figure

### Characteristics of salivary bacterial composition in the overall study population

We obtained 457 OTUs using a 97% homology cutoff. Our analysis included taxa from the six major phyla of the oral microbiome; we additionally filtered OTU taxa present in all participants in this analysis (14 phylum, 27 classes, 44 orders, 71 families, 159 genera, and 346 species). The *Firmicutes* and *Bacteroidetes* were two main abundant bacteria; the other most abundant bacteria at the family level were *Streptococcaceae* > *Prevotellaceae* > *Neisseriaceae* > *Veillonellaceae* > *Porphyromonadaceae* in the two groups. Compared with DM group, the relative abundance of *Gemella* in DN group was lower (2.38 ± 1.53 *vs.* 1.22 ± 0.57, *P* = 0.014). The relative abundance of bacteria from phylum to genus with > 1% abundance are presented in Fig. 1.

### Analysis of oral microbiota composition between DM group and DN group

**Fig. 2:**
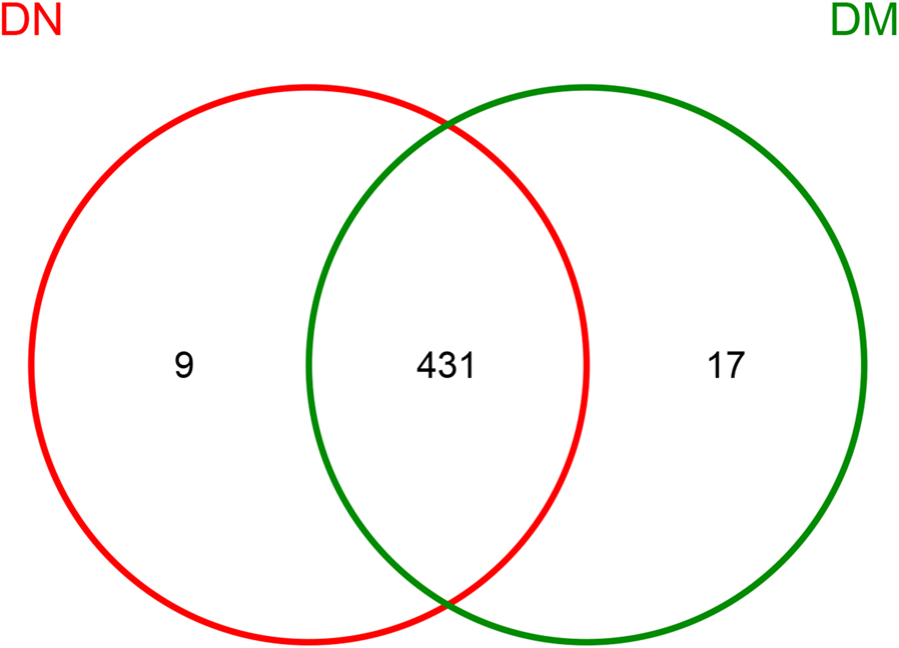
Shared and unique bacteria in patients with diabetes mellitus (DM) and diabetic nephropathy (DN). Different colors represent different groups. The overlap represents shared bacteria. There are 457 unique objects; of these, 431 appear in both groups

Venn diagram can intuitively illustrate the number of common and unique OTUs in a variety of samples. The DM group had the larger number of OTUs; 431 OTUs were common between the two groups. Seventeen specific OTUs in the DM group and 9 specific OTUs in the DN group are exhibited in the Venn diagram (Fig. 2) and the detailed information is shown in Additional file 1: Table S2. Thus, compared with the DN group, the DM group had more saliva flora specific OTUs. In DN group, the specific OTUs included *Peptococcus*, *Flavobacterium*, *Intestinimonas*, *Pelospora*, *Bacteroides*, *Fusobacterium*, *Ruminococcaceae_UCG-002*, *Megasphaera* at the genus level and *Mollicutes_RF9* at the order level.

**Table 2:** Results of binary logistic regression analysis showing risk factors for diabetic nephropathy

	Model 1			Model 2		
	OR	95% CI	*P* value	OR	95% CI	*P* value
Gemella	0	0.000–0.077	0.045	0.523	0.078–3.49	0.503
*Selenomonas spp.*	13.423	1.80–100.083	0.011	13.015	1.655–102.349	0.015

Model 1, univariate analysis. Model 2, multivariate analysis adjusting the effect of age, gender, and BMI

### Comparison of alpha-diversity of bacteria in the two groups

**Fig. 3:**
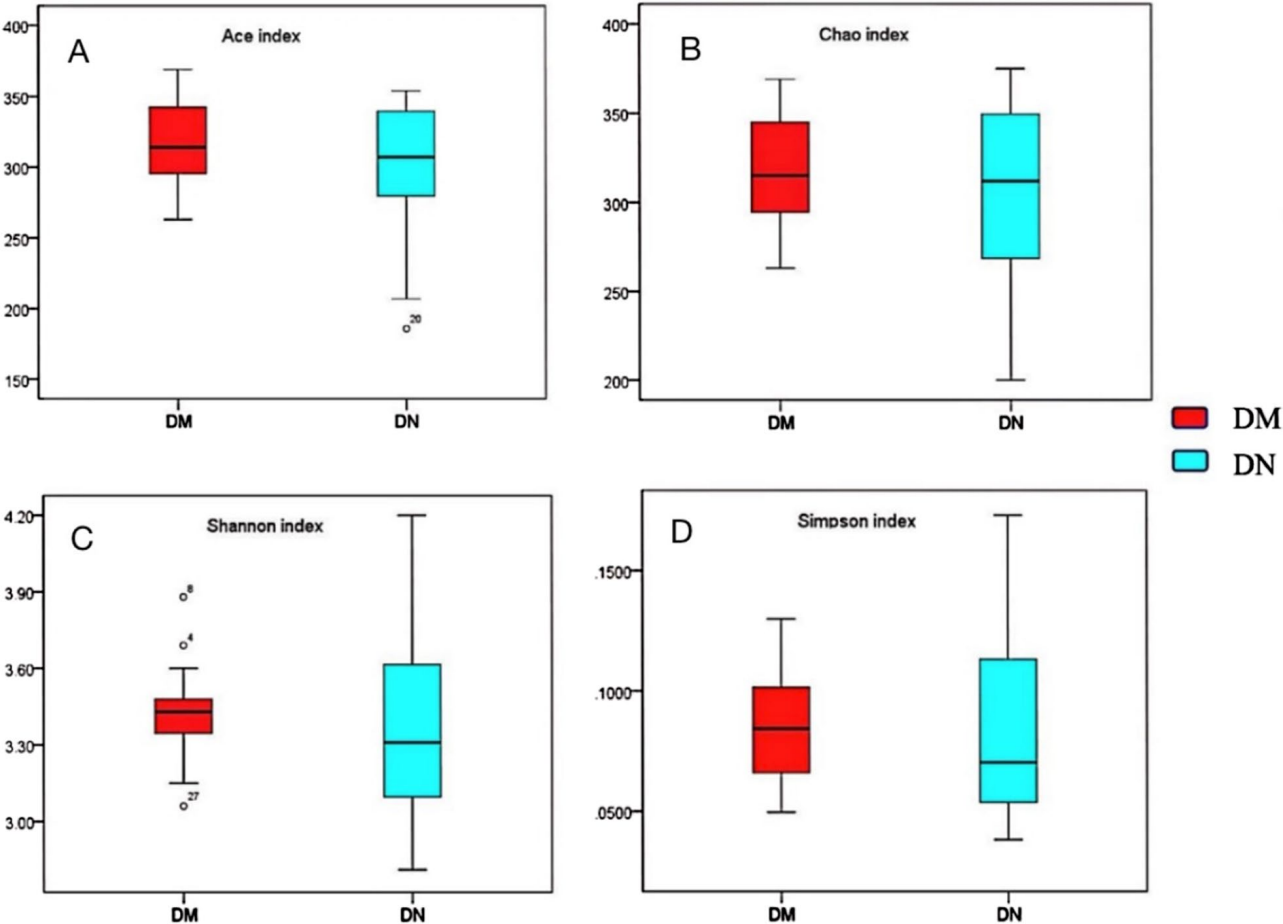
Alpha diversity of the saliva microbiome in patients with periodontitis and diabetes mellitus (DM) and patients with periodontitis and diabetic nephropathy (DN). (**a**) Ace index (*P* = 0.430) and (**b**) Chao index (*P* = 0.648) were abundance indices; (**c**) Shannon index (*P* = 0.468) and (**d**) Simpson index (*P* = 0.724) were diversity indices. The dots in Fig. 3C represent extreme values

Based on the OTU profile, alpha-diversity of Chao and ace indices were used to estimate community richness; Shannon and Simpson indices were used to estimate community diversity. The greater the Chao (*P* = 0.648) and Ace (*P* = 0.430) indices, the higher is the community richness. The greater the Shannon (*P* = 0.468) indices and Simpson indices (*P* = 0.724), the higher is the community diversity. We also observed a decreasing trend of species abundance and species evenness from DM group to DN group; however, the between-group difference in this respect was not statistically significant (*P* > 0.05) (Fig. 3).

### Comparison of beta-diversity of bacteria in the two groups

We sought to determine whether the overall microbiome composition differed according to presence or absence of DN. For this purpose, we conducted principal co-ordinate analysis (PCoA) of the oral microbiota profiles based on Weighted UniFrac distance measure; the results revealed a specific clustering with respect to diabetes mellitus or diabetic kidney disease along horizontal axis 1 (explaining 51.67% of the variation in the data; Additional file 1: Fig. S2).

### Significant differences of salivary microbiota between the two groups

**Fig. 4:**
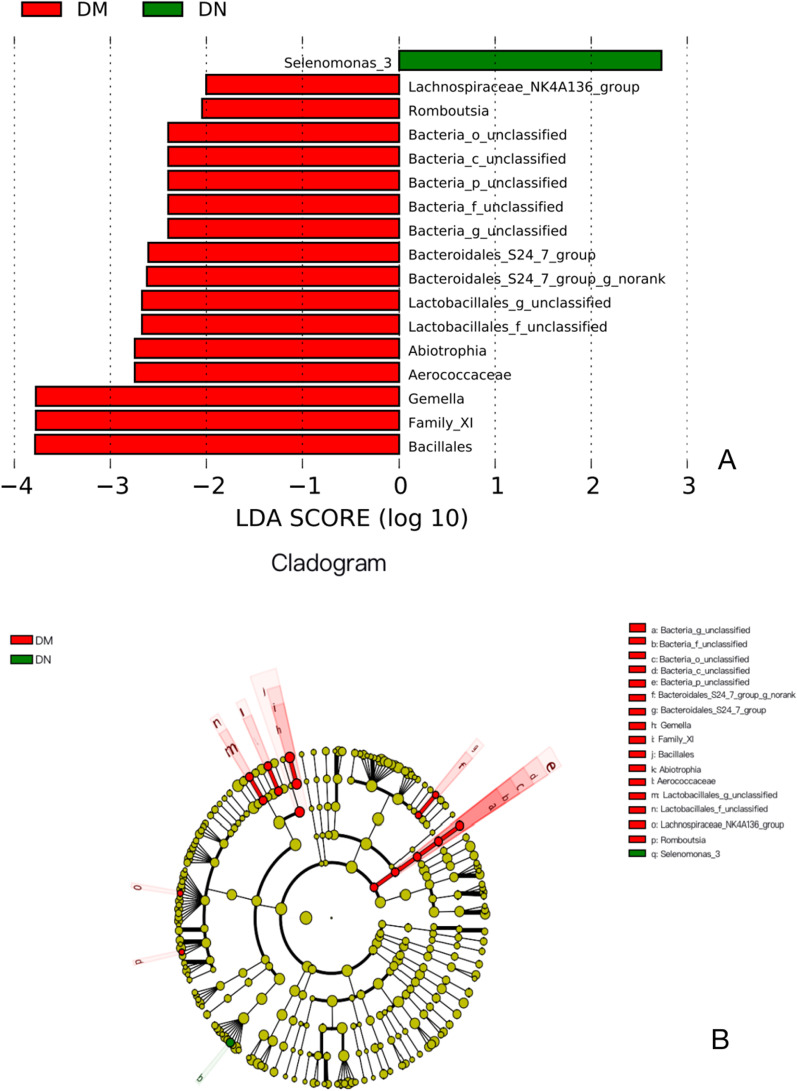
Partial bacterial taxa that differed significantly between patients with periodontitis and diabetes mellitus (DM group) and patients with periodontitis and diabetic nephropathy (DN group), according to linear discriminant analysis coupled with effect size (LEfSe). (**a**) Shows bacterial clades that are differentially abundant in DM group (red), and DN group (green). Clades in this graph were both statistically significant (p < 0.05) and had a linear discriminants (LDAs) score ≥ 2.0, which is considered a significant effect size. The circle from the inside to the outside in (**b**) represents the classification level from phylum to genera. The diameters of circles represent the relative abundance. Yellow indicates no significant difference; red and green dots represent significant differences in the pedigree for DM group and DN group (respectively)

Linear discriminant analysis (LDA) and an LDA value of 2.0 effect size (LEfSe) analyses were used to further characterize the differences in microbial communities between the two groups. Seventeen taxa were found to be significantly different between the two groups. LDA value of *Selenomonas spp.* was highest in the DN group while *Gemella* had the highest LDA value in the DM group (Fig. 4a). Significant changes in the *Bacteria_unclassified* and *Bacteria _unclassified class* were noted at all taxonomic levels down to genera (Fig. 4b).

**Fig. 5:**
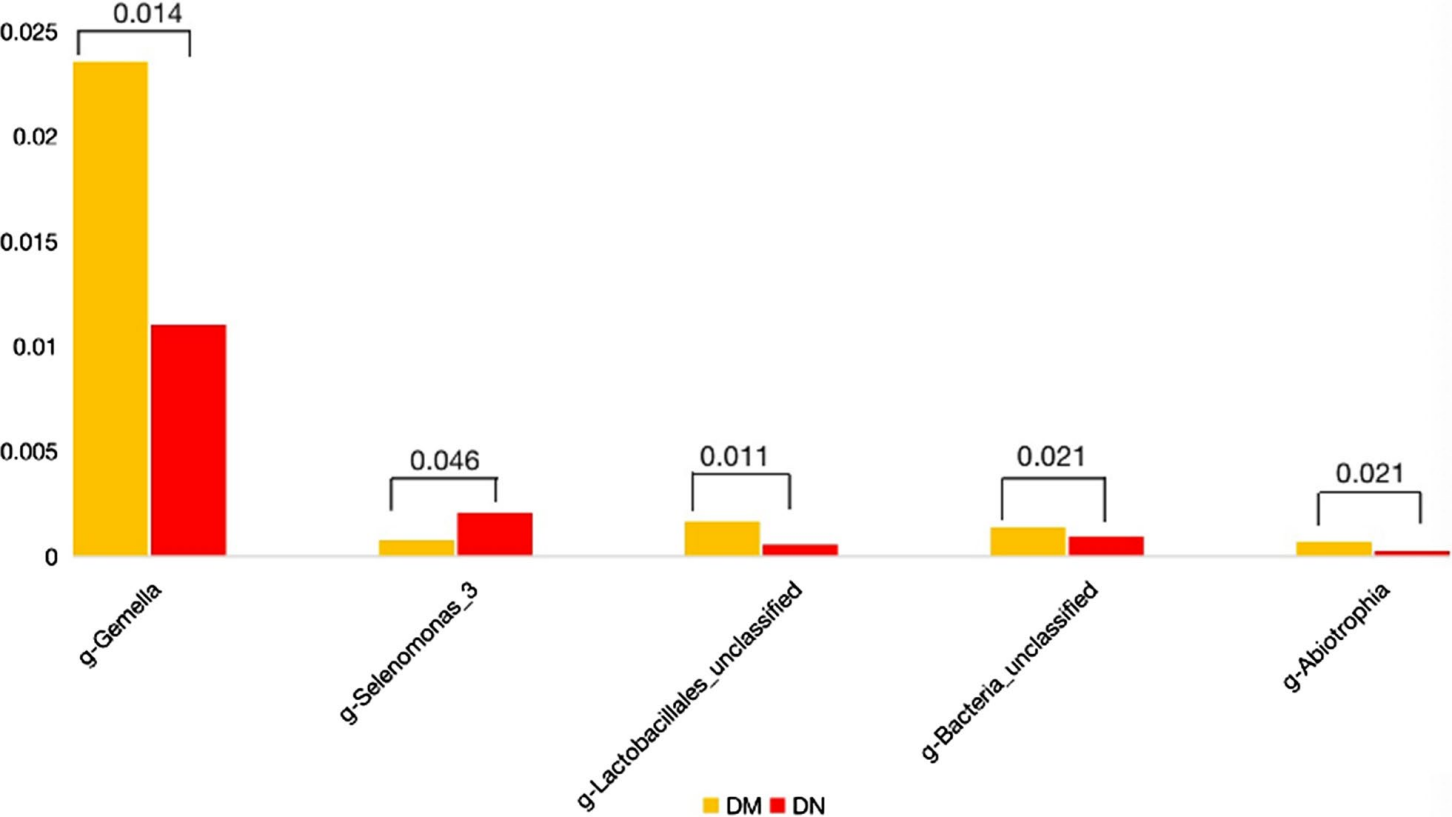
Mean relative abundance of the significantly altered microbiome in saliva among patients with periodontitis and diabetes mellitus (DM group) and patients with periodontitis and diabetic nephropathy (DN group). Between-group differences were assessed using the Mann–Whitney U test

Significant between-group differences were observed with respect to five bacterial genus (Mann–Whitney U test, *P* < 0.05). *Gemella*, *Lactobacillales_unclassified*, *Bacteria_unclassified,* and *Abiotrophia* were significantly more abundant in the DM group. On the contrary, *Selenomonas spp.* was detected more frequently in the DN group (Fig. 5).

### Risk factor for diabetic nephropathy

ROC curve analysis was used to assess the sensitivity for diagnosis of diabetic nephropathy. Since the area under the receiver operating curve (AUC) of *g_Selenomonas spp.* at the genus level was up to 0.713, the probability of true positive was higher (sensitivity: 0.867) and the probability of false positive was lower (specificity: 0.333). The results suggest that genus level *of Selenomonas spp.* may be considered as a potential sensitive marker of DN (Additional file 1: Fig. S3).

Binary logistic regression analysis was performed to identify the risk factors for DN (Table 2). Several variables (sex, age, BMI) were adjusted in the logistic regression models to minimize the impact of potential confounding factors. The increase in the relative abundance of *g-Selenomonas spp.* was related with DN (odds ratio: 13.015, 95% confidence interval:1.655–102.349, *P* = 0.015).

### Differences in the composition of functional genes

**Fig. 6:**
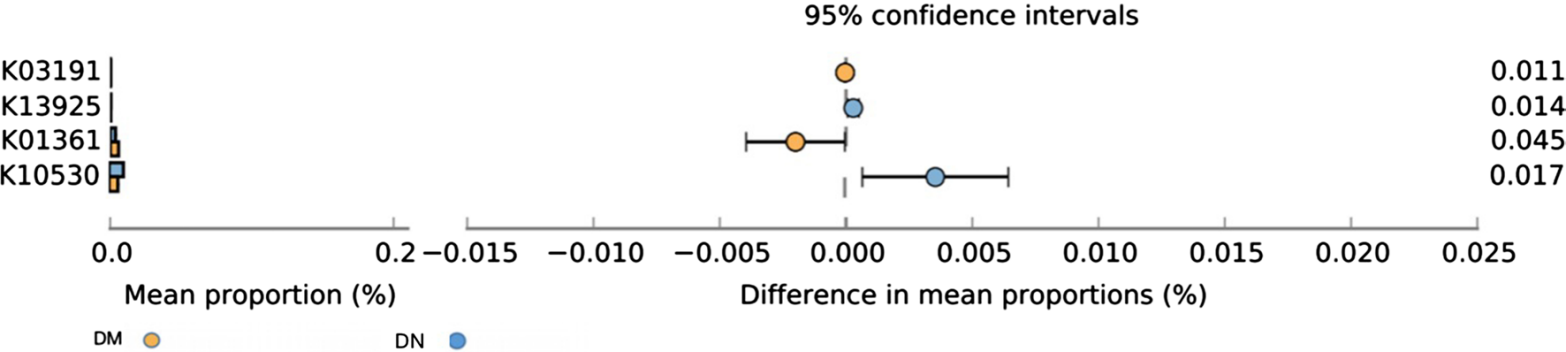
KEGG analysis of differences in the composition of functional genes

Compared with DM group, the functional gene expressions of acid-activated urea channel (K03191, *P* = 0.011)) and lactocepin (K01361, *P* = 0.045) were lower; on the contrary, plasmin and fibronetin-binding protein A (PfbA) (K13925, *P* = 0.014) and L-lactate oxidase (K10530, *P* = 0.017) were higher (Fig. 6).

## Discussion

To the best of our knowledge, no previous study has provided evidence of the potential role of oral microbiota in the development of renal complications of T2DM in patients with CP. In this study, we used 16 s rRNA gene sequencing method to analyze the differences of salivary microbial communities between DM patients with and without DN. In this study, we matched the two groups with respect to general characteristics, laboratory parameters, and periodontal parameters to rule out changes in oral flora caused by different periodontal conditions. Salivary samples were used for sequencing because of the convenience of collection. We found that patients in the DN group had significantly lower relative abundance of genus levels of *Gemella* (*P* = 0.014). Genus *Selenomonas spp.* can be used as a species feature to distinguish between DM patients with and without DN. Increased relative abundance of *Selenomonas spp.* may be associated with developing renal complications of T2DM with CP; this oral microbiome characteristic may serve as a potential marker of DN in patients with CP.

The DN group showed a tendency for decreased alpha diversity of oral microbiota as compared to the DM group. One study found higher alpha-diversity of the salivary microbiome in CP patients with DM compared to CP patients [6] without DM, while another study found no significant difference between CP patients with or without DM [7]. In both studies, DM treatment did not affect the alpha-diversity in CP patients with DM. However, in another study, CP patients undergoing peritoneal dialysis and CP patients with normal renal function showed no significant difference in diversity indices and species richness, but showed a tread of increasing diversity [14]. Collectively, these findings suggested that the progress of DM to DN in CP patients may decrease the alpha diversity of oral microbiota.

In this study, we found significant differences in the spectra of microbial communities between the DM and DN groups. The number of OTUs in the oral saliva flora of the DN group was slightly lower than that in the DM group. The composition of oral flora was different in the two groups. Compared with the DN group, *Gemella* was higher in the DM group. *Gemella* belongs to the *Firmicute* phylum, which is resident flora of the oral cavity. Previous studies have found increased relative abundance of *Gemella* in the subgingival plaque of patients with DM; it was categorized as an opportunistic pathogen [25]. *Gemella* belongs to the core microbial community in DM patients [26], and can cause severe localized and generalized infections as opportunistic pathogens for renal disease [27].

In our study, saliva levels of *Lactobacillales*_unclassified, Bacteria_unclassified, and *Abiotrophia* were significantly increased in the DM group. *Lactobacillus plantarum TN8* was shown to reduce cytokine IL-10/IL-12 ratio; in addition, oral administration of *Lactobacillus plantarum* TN8 was shown to improve the urinary function of obese rats [28]. Removal of 5/6^th^ of rat kidneys resulted in a significant decreases in the relative abundance of *Lactobacillacease* and *Prevotellaceae* families after 8 weeks [29]. In a study by Sato J et al., patients with T2DM showed significantly higher counts of *Lactobacillus* (facultative anaerobe) [30]. *Abiotrophia* were found in patients with chronic diseases such as periodontitis and dental caries [31]. *Abiotrophia* has been isolated from early dental plaques; the organism can produce hydrogen sulfide, which affects the plaque metabolism and ecology and causes periodontal disease [32]. The species are resident bacteria in the oral cavity and may cause peritonitis in dialysis patients [33]. Till date, the relationship between *Abiotrophia* and diabetes mellitus or diabetic nephropathy has not been elucidated.

The mean relative abundance of *Selenomonas spp.* was increased in the DN group; *Selenomonas spp.* may be the specific flora in the saliva of patients with DN. This was consistent with a previous study that found significant enrichment of *Selenomonas* in the subgingival flora of peritoneal dialysis patients with periodontitis [14]. *Selenomonas* belongs to phylum *Firmicute*; it is a G-negative, anaerobic, crescent-shaped bacterium. *Selenomonas* was reported to be associated with peri-implant health [34]. A previous study found a positive correlation between probing depth and the levels of *Selenomonas* [35]. *Selenomonas* species are resident bacteria in the digestive system, which showed significantly higher abundance in DM group than in non-DM group [36]; thus, genus *Selenmonas* have repeatedly been associated with periodontitis[37].

Oral saliva has a low relative abundance of *Selenomonas spp.*; however, the results of LEfSe in our study showed that *Selenomonas spp.* is a special bacterium of oral saliva flora in the DN group. ROC curve analysis showed that *Selenomonas spp.* may be a potential sensitive marker of DN. On binary logistic regression analysis, increased relative abundance of *Selenomonas spp.* was related with DN in patients with DM, after controlling for potential confounding variables. In our study, the levels of urea nitrogen in the DN group were a little higher than those in the DM group although without statistical significance. Increasing urea concentration during chronic kidney disease can lead to alterations in the intestinal flora [38]. Therefore, higher urea nitrogen level might suggest a possible imbalance of intestinal tract bacteria in patients with DN. Thus, *Selenomonas spp.* is a potential biomarker of DN; increase in its relative abundance suggests a higher risk of kidney complications in diabetic patients with CP.

KEGG analysis revealed that PfbA and L-lactate oxidase were significantly higher in the DN group. PfbA, mainly expressed on the pneumococcal cell surface, can bind to human serum proteins and is an important factor in the development of pneumococcal infections [39]. Further studies are required to investigate how PfbA functions during the progression of DM to DN. Lactate oxidase is related to oxidative stress and produces a large amount of H_2_O_2_, which can lead to islets β cell damage and induce glomerular mesangial cell apoptosis; in addition, oxidative stress can aggravate renal tissue damage [40]. Therapeutic strategies against oxidative stress to reduce the inflammatory response of renal tissue are widely applied in the treatment of early DN[41]. Therefore, the upregulation of L-lactate oxidase may contribute to the progression of DM to DN.

Some limitations of this study should be considered while interpreting the results. The small number of subjects in each group was a key limitation. Only 15 subjects with controlled T2DM and 15 subjects with DN were included. Second, this was an observational study; we did not determine the impact of changes in oral flora after periodontal treatment on the occurrence and development of diabetic nephropathy. Third, we did not include non-diabetic patients with periodontitis in this study. Finally, due to incomplete data records, the plaque index and gingival index were not analyzed. The above limitations can be overcome by increasing the sample size and by examining the effect of periodontal treatment in future studies.

## Conclusions

In conclusion, characterization of the differences of oral mircoflora between DN patients and DM patients without nephropathy can provide novel insights about the early treatment and prevention of DN. Increase in the relative abundance of genera *Selenomonas spp.* was associated with a higher risk of DN. *Selenomonas spp.* maybe a potential marker of DN. Our findings suggest that the changes in the composition of oral microbiome may be related with DN. Future studies should include a larger sample size and perform metagenome sequencing to provide more comprehensive results.

## Supplementary Information

**Additional file 1.** **Supplementary Figure 1.** Flowchart of the included patients. **Supplementary Figure 2.** Weighted UniFrac principal co-ordinate analysis was used to compare community phylogenetic composition in the samples of patients with periodontitis and diabetes mellitus (DM group, red color) and patients with periodontitis and diabetic nephropathy (DN group, blue color). **Supplementary Figure 3.** ROC curve showing g-Selenomonas spp. as a sensitive indicator for the diagnosis of diabetic nephropathy. **Supplementary Table 1.** Data of periodontal examination. **Supplementary Table 2.** Specific taxa for DM and DN.

## Data Availability

The datasets generated and analyzed during the current study are not publicly available due to none of the data types requiring uploading to a public repository but are available from the corresponding author on reasonable request.

## Abbreviations

DN: Diabetic nephropathy
DM: Diabetes mellitus
ROC: Receiver operating characteristic
AUC: Area under the receiver operating curve
CP: Chronic periodontitis
T2DM: Type 2 diabetes mellitus
CKD: Chronic kidney disease
ESRD: End-stage renal disease
BMI: Body mass index
FBG: Fasting blood glucose
HbA1c: Glycated hemoglobin
BI: Bleeding index
PD: Probing depth
CAL: Clinical attachment loss
CEJ: Cemento-enamel junction
ACR: Albumin/creatinine ratio
eGFR: Estimated glomerular filtration rate
OTUs: Operational taxonomic units
PCoA: Principle coordinate analysis
LEfSe: Linear discriminant analysis effect size
LDA: Linear discriminant analysis
KEGG: Kyoto encyclopedia of genes and genomes
