# Supplementary material for: Difference in oral microbial composition between chronic periodontitis patients with and without diabetic nephropathy
Source: BMC Oral Health. 2022 Jan 16;22:12. doi: 10.1186/s12903-021-01985-3 (PMC8762855; doi:10.1186/s12903-021-01985-3)
Supplement: Supplementary file 1 — Additional file 1. Supplementary Figure 1. Flowchart of the included patients. Supplementary Figure 2. Weighted UniFrac principal co-ordinate analysis was used to compare community phylogenetic composition in the samples of patients with periodontitis and diabetes mellitus (DM group, red color) and patients with periodontitis and diabetic nephropathy (DN group, blue color). Supplementary Figure 3. ROC curve showing g-Selenomonas spp. as a sensitive indicator for the diagnosis of diabetic nephropathy. Supplementary Table 1. Data of periodontal examination. Supplementary Table 2. Specific taxa for DM and DN. [file 12903_2021_1985_MOESM1_ESM.doc]

**Difference in oral microbial composition between chronic periodontitis patients with and without diabetic nephropathy**

**Running title: Oral microbiota in DN patients**

Dongxue Zhang^1^, Wenyan Liu^2^, Li Peng^3^, Haiyan Wang^1^, Mei Lin^1^, Yufeng Li^4*^, Zuomin Wang^1*^

^1^ Department of Stomatology, Beijing Chao-Yang Hospital, Capital Medical University, Beijing, 100020, China

^2^ Department of Stomatology, Beijing Luhe Hospital, Capital Medical University, Beijing, 101149, China

^3^ Department of Stomatology, The Third People’s Hospital of Datong City, Datong, 037008, China

^4^ Department of Endocrinology, Beijing Friendship Hospital Pinggu Campus,

Capital Medical University, Beijing, 101200, China

***Corresponding Author:**

Yufeng Li

Department of Endocrinology, Beijing Friendship Hospital Pinggu Campus, Capital Medical University, No.59 Xinping North Road, Pinggu District, Beijing, 101200, China.

Tel./ Fax: +8601089978790; E-mail: [doctorlyf@126.com](mailto:doctorlyf@126.com)

Zuomin Wang

Department of Stomatology, Beijing Chao-Yang Hospital, Capital Medical University, No. 8 South Gongti Road, Chao Yang District, Beijing, 100020, China,

Tel./ Fax: +8601085231492; E-mail: [wzuomin@sina.cn](mailto:wzuomin@sina.cn)

***Supplementary Materials***

**Supplementary Figure Legends**

**Supplementary Figure 1** Flowchart of the included patients.

**Supplementary Figure 2** Weighted UniFrac principal co-ordinate analysis was used to compare community phylogenetic composition in the samples of patients with periodontitis and diabetes mellitus (DM group, red color) and patients with periodontitis and diabetic nephropathy (DN group, blue color).

**Supplementary Figure 3** ROC curve showing g-*Selenomonas spp.* as a sensitive indicator for the diagnosis of diabetic nephropathy (area under the curve: 0.713).

**Supplementary Table 1** Data of periodontal examination.

| **Group** | **PD mean** | **BI mean** | **CAL mean** | **PD≥4 mm (%)** | **PD≥5 mm (%)** | **CAL≥3 mm (%)** | **CAL≥4 mm (%)** | **Remaining teeth numbers** |
| --- | --- | --- | --- | --- | --- | --- | --- | --- |
| DN | 3.63 | 3.61 | 5.11 | 33.10 | 18.71 | 73.91 | 56.52 | 23 |
| DN | 4.66 | 3.29 | 6.68 | 68.94 | 40.91 | 84.09 | 40.91 | 22 |
| DN | 3.55 | 3.68 | 6.35 | 35.88 | 10.69 | 88.10 | 26.19 | 22 |
| DN | 2.73 | 2.77 | 4.09 | 10.92 | 0.76 | 81.82 | 45.45 | 22 |
| DN | 4.02 | 4 | 5.42 | 60.32 | 19.84 | 100.00 | 16.67 | 21 |
| DN | 2.56 | 1.54 | 3.52 | 4.71 | 0.00 | 71.43 | 53.57 | 28 |
| DN | 2.72 | 2.46 | 6.26 | 13.89 | 0.00 | 91.67 | 88.33 | 12 |
| DN | 4.04 | 3.32 | 9.07 | 55.67 | 32.97 | 100.00 | 8.06 | 31 |
| DN | 5.13 | 3.56 | 9.46 | 71.68 | 45.26 | 77.78 | 22.22 | 9 |
| DN | 4.67 | 3.64 | 7.72 | 78.58 | 50.60 | 89.29 | 74.29 | 28 |
| DN | 2.8 | 3.04 | 3.26 | 10.90 | 0.00 | 67.31 | 58.85 | 26 |
| DN | 2.43 | 2.83 | 2.91 | 9.42 | 4.35 | 39.13 | 31.74 | 23 |
| DN | 2.51 | 2.89 | 2.65 | 9.19 | 1.62 | 41.94 | 21.94 | 30 |
| DN | 2.61 | 2.31 | 2.98 | 11.80 | 3.47 | 52.08 | 51.25 | 24 |
| DN | 2.63 | 1.95 | 3.45 | 11.36 | 5.30 | 48.84 | 34.10 | 22 |
| DM | 4.24 | 3.81 | 5.78 | 73.43 | 38.46 | 91.49 | 62.77 | 24 |
| DM | 2.20 | 2.78 | 5.37 | 16.70 | 0.00 | 32.50 | 9.00 | 20 |
| DM | 2.79 | 2.32 | 4.19 | 15.33 | 3.33 | 68.00 | 52.00 | 25 |
| DM | 3.26 | 3.95 | 5.37 | 29.34 | 4.19 | 92.86 | 33.93 | 28 |
| DM | 3.96 | 3.5 | 7.42 | 58.38 | 32.21 | 88.00 | 14.00 | 25 |
| DM | 2.76 | 2.77 | 2.80 | 15.29 | 2.35 | 46.43 | 38.21 | 28 |
| DM | 3.62 | 3.9 | 4.12 | 45.11 | 17.39 | 91.94 | 51.61 | 31 |
| DM | 2.78 | 3.89 | 4.59 | 15.74 | 2.78 | 80.56 | 52.78 | 18 |
| DM | 5.44 | 3.79 | 7.46 | 77.60 | 71.20 | 80.95 | 23.81 | 21 |
| DM | 2.45 | 2.26 | 3.45 | 8.69 | 0.62 | 50.00 | 32.22 | 27 |
| DM | 4.95 | 4.00 | 5.14 | 75.60 | 55.36 | 98.21 | 79.64 | 28 |
| DM | 2.91 | 3.52 | 3.46 | 15.67 | 4.86 | 62.90 | 12.90 | 31 |
| DM | 2.49 | 2.28 | 2.57 | 3.33 | 0.00 | 38.33 | 0.10 | 30 |
| DM | 3.89 | 3.94 | 6.28 | 60.19 | 21.30 | 94.44 | 25.00 | 18 |
| DM | 2.98 | 2.94 | 4.32 | 16.67 | 3.13 | 78.13 | 53.13 | 16 |

PD, pocket probing depth; BI, bleeding index; CAL, clinical attachment level, **PD≥4 mm%,** Six locations were detected in each tooth, PD≥4 mm%= all locations with PD≥4 mm/(the number of teeth×6)

**Supplementary Table 2** Specific taxa for DM and DN

| Group | OTU ID |  |
| --- | --- | --- |
| DM | OTU10 | d__Bacteria; p__Bacteroidetes; c__Bacteroidia; o__Bacteroidales; f__Porphyromonadaceae |
| DM | OTU86 | d__Bacteria; p__Bacteroidetes; c__Flavobacteriia; o__Flavobacteriales; f__Flavobacteriaceae; g__Capnocytophaga; s__Capnocytophaga_haemolytica |
| DM | OTU90 | d__Bacteria; p__Firmicutes; c__Clostridia; o__Clostridiales; f__Peptostreptococcaceae; g__Paeniclostridium |
| DM | OTU136 | d__Bacteria; p__Bacteroidetes; c__Bacteroidia; o__Bacteroidales; f__Bacteroidales_S24-7_group; g__norank; s__uncultured_bacterium |
| DM | OTU169 | d__Bacteria; p__Fusobacteria; c__Fusobacteriia; o__Fusobacteriales; f__Leptotrichiaceae; g__Leptotrichia |
| DM | OTU218 | d__Bacteria; p__Fusobacteria; c__Fusobacteriia; o__Fusobacteriales; f__Leptotrichiaceae; g__Leptotrichia; s__uncultured_bacterium |
| DM | OTU228 | d__Bacteria; p__Proteobacteria; c__Alphaproteobacteria; o__Rhizobiales; f__Phyllobacteriaceae |
| DM | OTU257 | d__Bacteria; p__Bacteroidetes; c__Bacteroidia; o__Bacteroidales; f__Bacteroidales_S24-7_group; g__norank; s__uncultured_bacterium |
| DM | OTU270 | d__Bacteria; p__Bacteroidetes; c__Bacteroidia; o__Bacteroidales; f__Porphyromonadaceae; g__Porphyromonas |
| DM | OTU335 | d__Bacteria; p__Bacteroidetes; c__Bacteroidia; o__Bacteroidales; f__Bacteroidales_S24-7_group; g__norank; s__uncultured_bacterium |
| DM | OTU343 | d__Bacteria; p__Firmicutes; c__Clostridia; o__Clostridiales; f__Lachnospiraceae; g__Lachnospiraceae_NK4A136_group |
| DM | OTU355 | d__Bacteria; p__Firmicutes; c__Clostridia; o__Clostridiales; f__Lachnospiraceae |
| DM | OTU378 | d__Bacteria; p__Actinobacteria; c__Actinobacteria; o__Corynebacteriales; f__Corynebacteriaceae; g__Corynebacterium; s__Corynebacterium_sp._canine_oral_taxon_423 |
| DM | OTU420 | d__Bacteria; p__Bacteroidetes; c__Bacteroidia; o__Bacteroidales; f__Prevotellaceae; g__Alloprevotella; s__uncultured_Bacteroidales_bacterium |
| DM | OTU435 | d__Bacteria; p__Spirochaetae; c__Spirochaetes; o__Spirochaetales; f__Spirochaetaceae; g__Spirochaeta_2; s__Spirochaeta_sp._canine_oral_taxon_314 |
| DM | OTU439 | d__Bacteria; p__Bacteroidetes; c__Bacteroidia; o__Bacteroidales; f__Bacteroidales_S24-7_group; g__norank; s__uncultured_bacterium |
| DM | OTU450 | d__Bacteria; p__Fusobacteria; c__Fusobacteriia; o__Fusobacteriales; f__Leptotrichiaceae; g__Streptobacillus; s__Streptobacillus_hongkongensis |
| DN | OTU47 | d__Bacteria; p__Firmicutes; c__Clostridia; o__Clostridiales; f__Peptococcaceae; g__Peptococcus |
| DN | OTU84 | d__Bacteria; p__Bacteroidetes; c__Flavobacteriia; o__Flavobacteriales; f__Flavobacteriaceae; g__Flavobacterium |
| DN | OTU142 | d__Bacteria; p__Tenericutes; c__Mollicutes; o__Mollicutes_RF9; f__norank; g__norank |
| DN | OTU167 | d__Bacteria; p__Firmicutes; c__Clostridia; o__Clostridiales; f__Ruminococcaceae; g__Intestinimonas |
| DN | OTU202 | d__Bacteria; p__Firmicutes; c__Clostridia; o__Clostridiales; f__Syntrophomonadaceae; g__Pelospora; s__Syntrophomonadaceae_genomosp._P1 |
| DN | OTU273 | d__Bacteria; p__Bacteroidetes; c__Bacteroidia; o__Bacteroidales; f__Bacteroidaceae; g__Bacteroides; s__Prevotella_zoogleoformans |
| DN | OTU309 | d__Bacteria; p__Fusobacteria; c__Fusobacteriia; o__Fusobacteriales; f__Fusobacteriaceae; g__Fusobacterium; s__Fusobacterium_necrophorum_subsp._necrophorum |
| DN | OTU323 | d__Bacteria; p__Firmicutes; c__Clostridia; o__Clostridiales; f__Ruminococcaceae; g__Ruminococcaceae_UCG-002; s__uncultured_bacterium |
| DN | OTU447 | d__Bacteria; p__Firmicutes; c__Negativicutes; o__Selenomonadales; f__Veillonellaceae; g__Megasphaera; s__uncultured_bacterium |


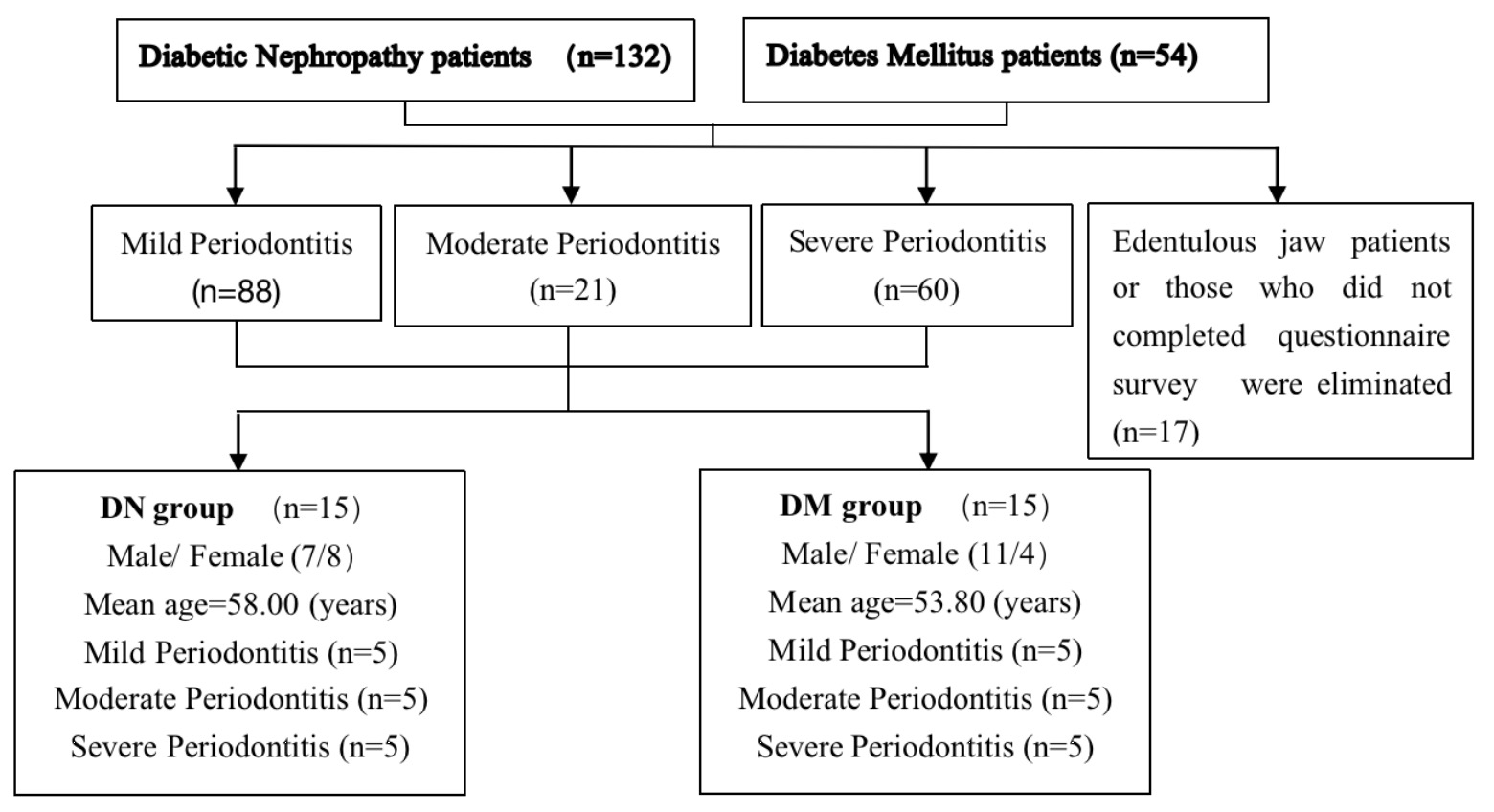


**Supplementary Figure 1**

**
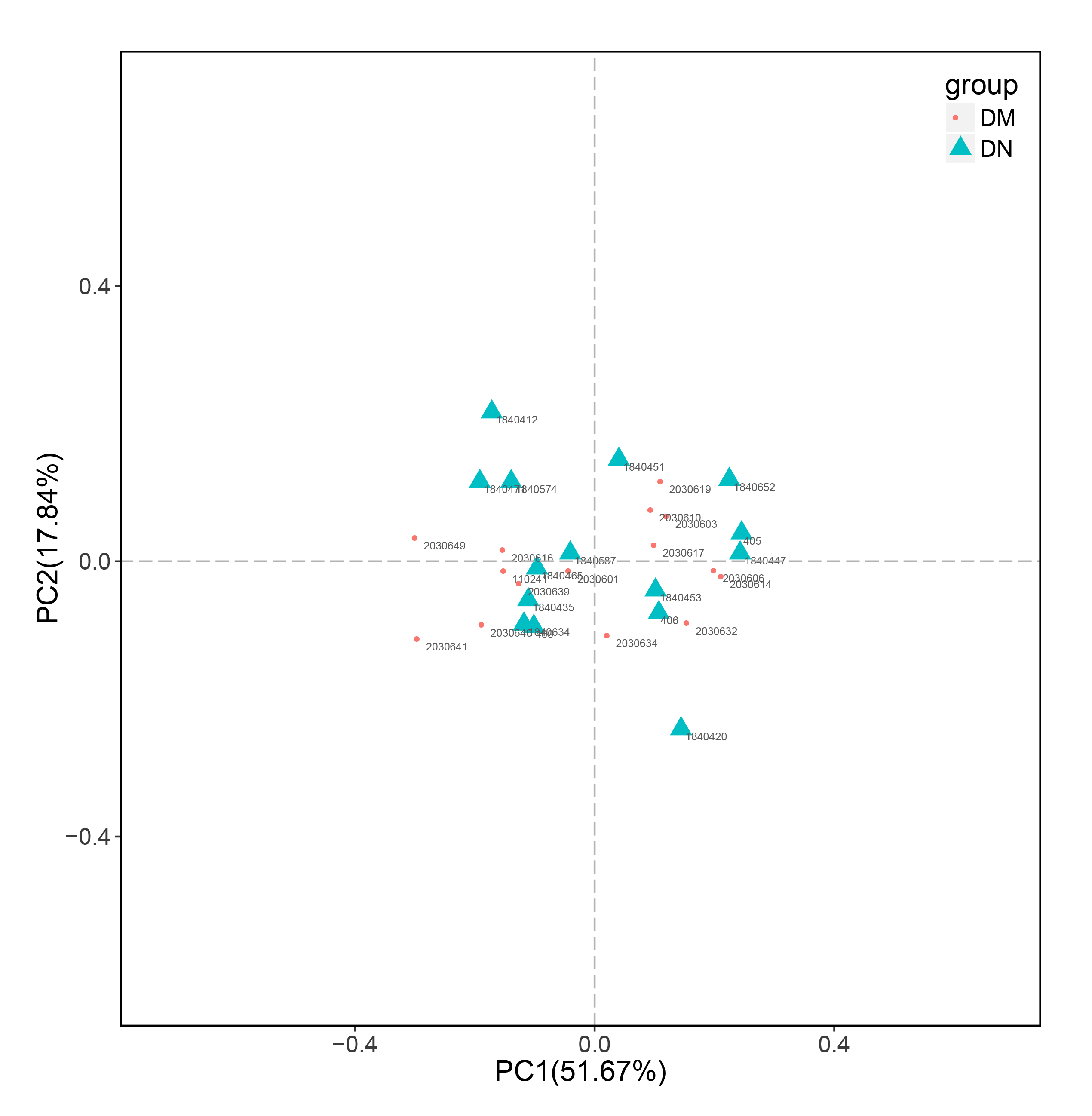
**

**Supplementary Figure 2**

**
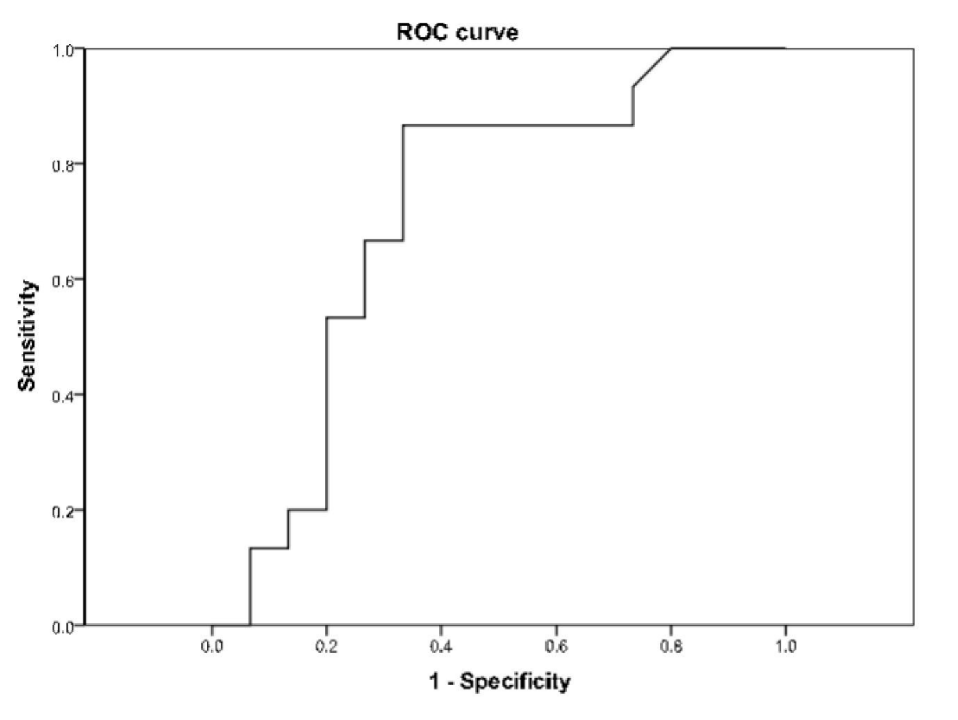
**

**Supplementary Figure 3**
